# Case Report: All that glitters is not cancer; perihepatic hibernoma with fluctuating FDG uptake on PET/CT

**DOI:** 10.3389/fnume.2024.1477467

**Published:** 2024-10-23

**Authors:** Amaila Ramzan, Amarjot Chander, Thomas Westwood, Mark Elias, Prakash Manoharan

**Affiliations:** Radiology Department, The Christie, NHS Foundation Trust, Manchester, United Kingdom

**Keywords:** hibernoma, perihepatic, FDG PET/CT, brown fat, ambient (atmospheric) temperature

## Abstract

Hibernomas are rare brown fat tumors that garnered attention in the literature with the increasing use of [^18^F] Fluorodeoxyglucose Positron Emission Tomography/Computed Tomography ([^18^F] FDG PET/CT) for the staging workup and follow-up of solid malignancies. Despite being benign tumors, they exhibit high metabolic activity due to their thermogenic nature, leading to significant radiotracer uptake on functional imaging. This can pose a challenge in differentiating them from the malignant lesions, especially the fat-containing malignancies such as liposarcoma. Hibernomas are typically found in the thigh, shoulder, back, and neck. Here, we present a unique case of Hibernoma in a patient undergoing PET/CT for melanoma follow-up in an unusual perihepatic location. To the best of the authors’ knowledge, this represents the first reported case of a perihepatic hibernoma in the literature. The report also offers a literature review on hibernomas, including the influence of ambient temperature on their metabolism, diagnostic challenges, management strategies, and reports of hibernomas detected on functional imaging with a range of radiotracers. These observations could serve as a valuable clue in identifying hibernomas, potentially aiding in avoiding unnecessary biopsies or resections.

## Introduction

Fluorodeoxyglucose Positron Emission Tomography/Computed Tomography (FDG PET/CT) is increasingly employed in diagnosing and staging solid tumors. High FDG uptake above the background may suggest malignancy; however, factors such as artifacts, infection, and inflammation can also increase FDG uptake, necessitating a comprehensive understanding of potential false-positive results in PET/CT studies (1).

Hibernoma, a benign tumor originating from fetal brown fat remnants, is known for its avid FDG uptake, causing potential confusion in patients undergoing imaging for cancer assessment. While the fatty appearance of the lesion on CT imaging aids in differentiation from solid or necrotic tumors, distinguishing it from fat-containing tumors like liposarcoma presents a more significant challenge. This distinction is crucial for PET/CT reporters to prevent erroneous positive findings (2).

This report seeks to present a rare case of Hibernoma in the perihepatic location, an unprecedented occurrence in the existing literature. The aim is to raise awareness among readers about this unusual brown fat tumor, provide background information, highlight common locations, and, through this case, illustrate an infrequent location to enhance the accuracy of PET/CT reporting.

Additionally, a brief literature review is included to share insights from researchers on the diagnosis, characterization, and management of Hibernomas. Reports of hibernomas identified on imaging with newer PET/CT radiotracers, such as ^68^Gallium-DOTATAE (^68^Ga-DOTATATE), ^68^Gallium-Prostate specific membrane antigen (^68^Ga-PSMA), and Gallium-68 fibroblast activation protein inhibitor ([^68^Ga]Ga-FAPI), have also been shared. Furthermore, a novel perspective is introduced, suggesting the consideration of changes in Standardized Uptake Values (SUV) based on ambient temperatures during malignancy follow-up as a potential diagnostic approach for Hibernomas (3, 4).

## Case description

A 40-year-old male patient with a known history of type 2 diabetes, high cholesterol, and gout presented in May 2013 with a skin lesion on the right upper anterior chest wall and enlarged right axillary lymph nodes. Histological assessment confirmed stage IIIB malignant melanoma, which was successfully treated through local excision and axillary clearance. However, in July 2014, a recurrence developed in the left anterior chest, which was once again managed with local excision. Due to a high risk of further recurrence, the patient was placed under surveillance.

**Figure 1:**
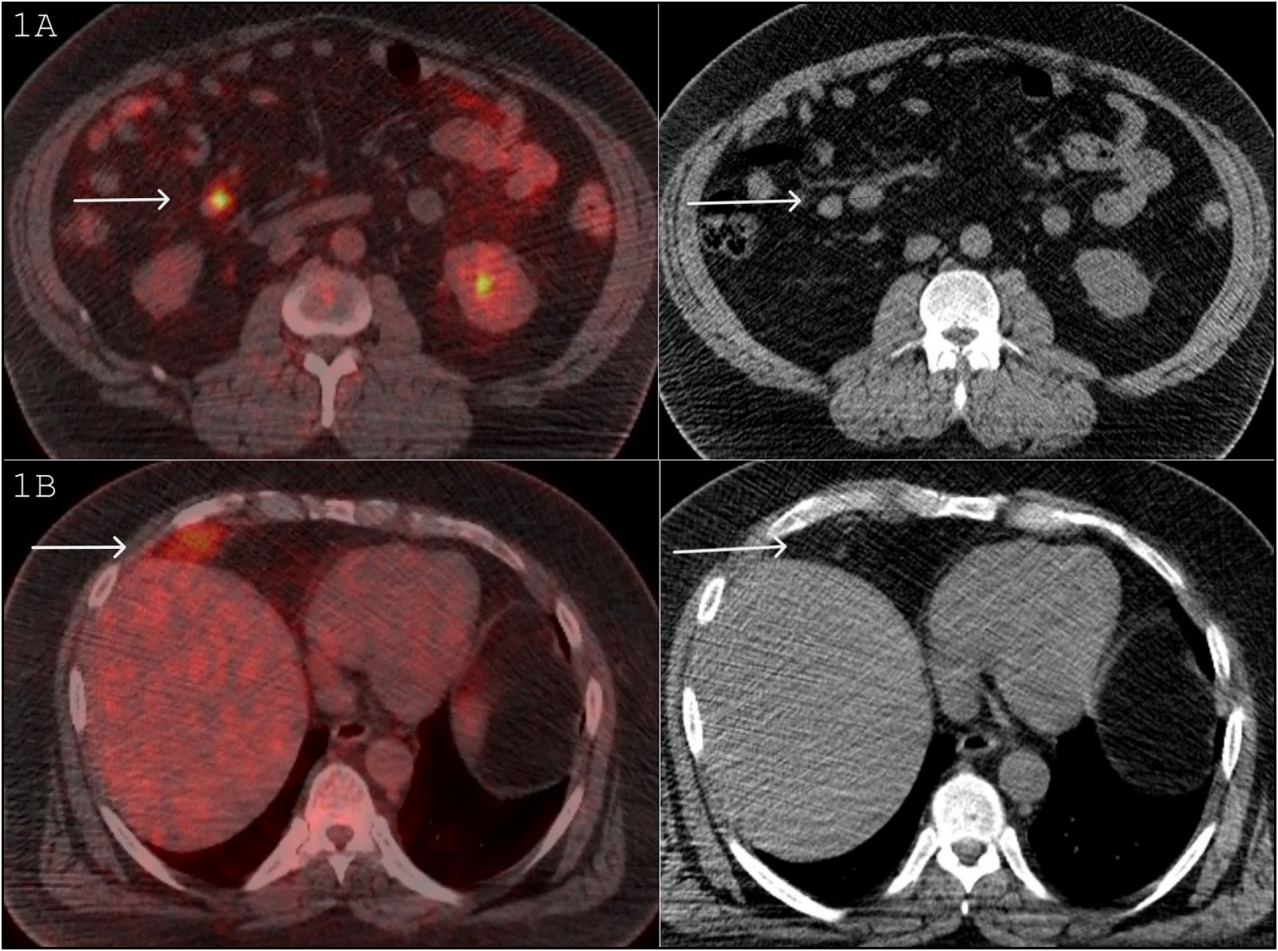
**(A)** FDG PET/CT November 2015: A 2.1 × 1.6 cm FDG avid lesion on the right side of the mesentery (SUV max 9.5, background hepatic SUV max 3.5), raising concerns for an abdominal melanoma metastasis. **(B)** FDG PET/CT November 2015: FDG avid mass in the right perihepatic location (SUV max 6.5). On the CT component, this mass is fat density and contains a large vessel.

The patient remained in remission for a year and a half until November 2015, when he developed new iron deficiency anemia and bone pain. To assess potential disease recurrence, an FDG PET/CT was performed, which revealed a 2.1 × 1.6 cm FDG avid lesion on the right side of the mesentery (SUV max 9.5, background hepatic SUV max 3.5), raising concerns for abdominal melanoma metastasis (Figure 1A).

The study also revealed another FDG avid mass in the right perihepatic location (SUV max 6.5), flattening the adjacent hemidiaphragm (Figure 1B). On the CT component of the study, this mass was of predominantly fat density with some heterogeneity and contained a large vessel (Figure 1B). The potential differential diagnoses included melanoma metastasis, liposarcoma, lipoma, angiomyolipoma, and Hibernoma. Retrospectively, a review of a CT scan in 2013 showed that this lesion was present then as well and had been stable in size and CT appearance. The patient commenced treatment with Pembrolizumab.

**Figure 2:**
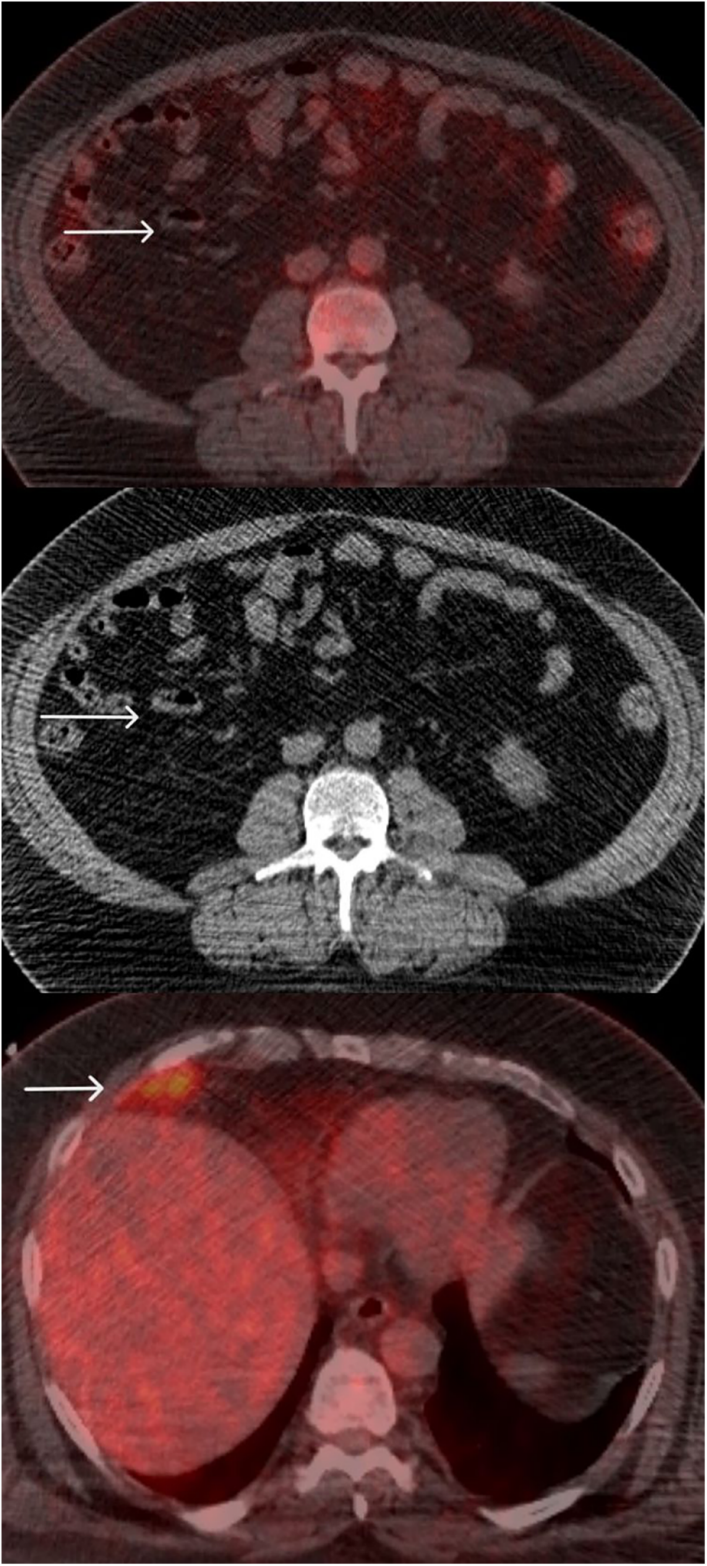
FDG PET/CT June 2016: follow-up PET/CT after 7 cycles of immunotherapy shows a positive response in the mesenteric node (Top image), but the perihepatic lesion is morphologically stable. The FDG uptake reduced from SUV max 6.5 to 5.3 (background hepatic SUV max 4).

A follow-up PET/CT after seven cycles of immunotherapy in June 2016 documented a positive response in the mesenteric node, but the perihepatic lesion remained morphologically stable. The FDG uptake, however, was reduced from SUV max 6.5 to 5.3 (background hepatic SUV max 4) (Figure 2).

**Figure 3:**
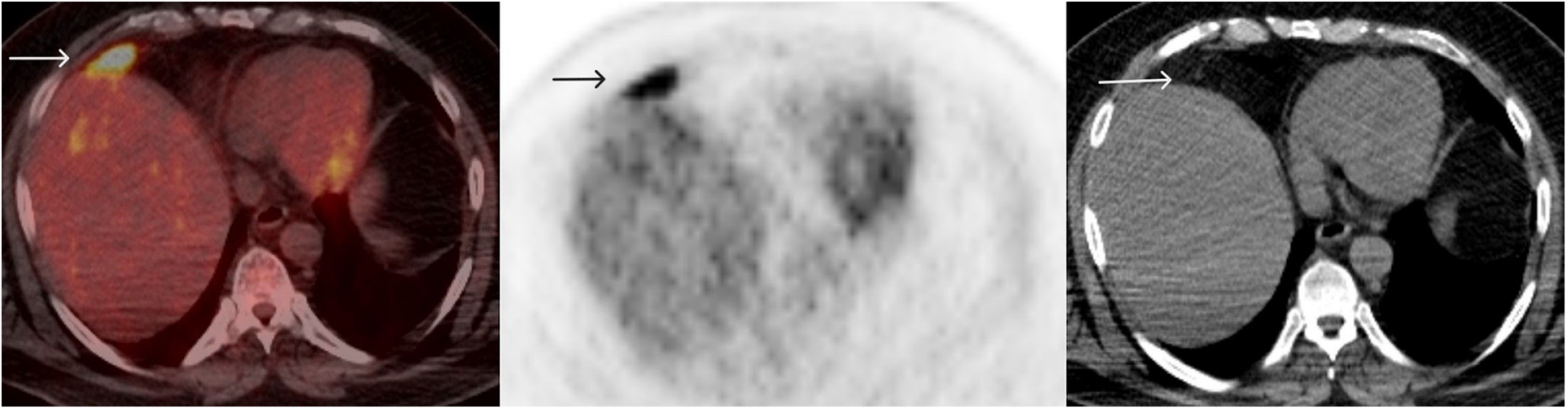
FDG PET/CT December 2018: after 12 cycles of immunotherapy, FDG uptake in the perihepatic lesion increased to SUV max 7.8 (background hepatic SUV max 2.8).

The patient continued with further immunotherapy, completing 12 cycles over 24 months by December 2017. The PET/CT in 2018 indicated a complete response in the mesenteric nodule, but the FDG uptake in the perihepatic lesion increased this time to SUV max 7.8 (background hepatic SUV max 2.8) (Figure 3).

**Figure 4:**
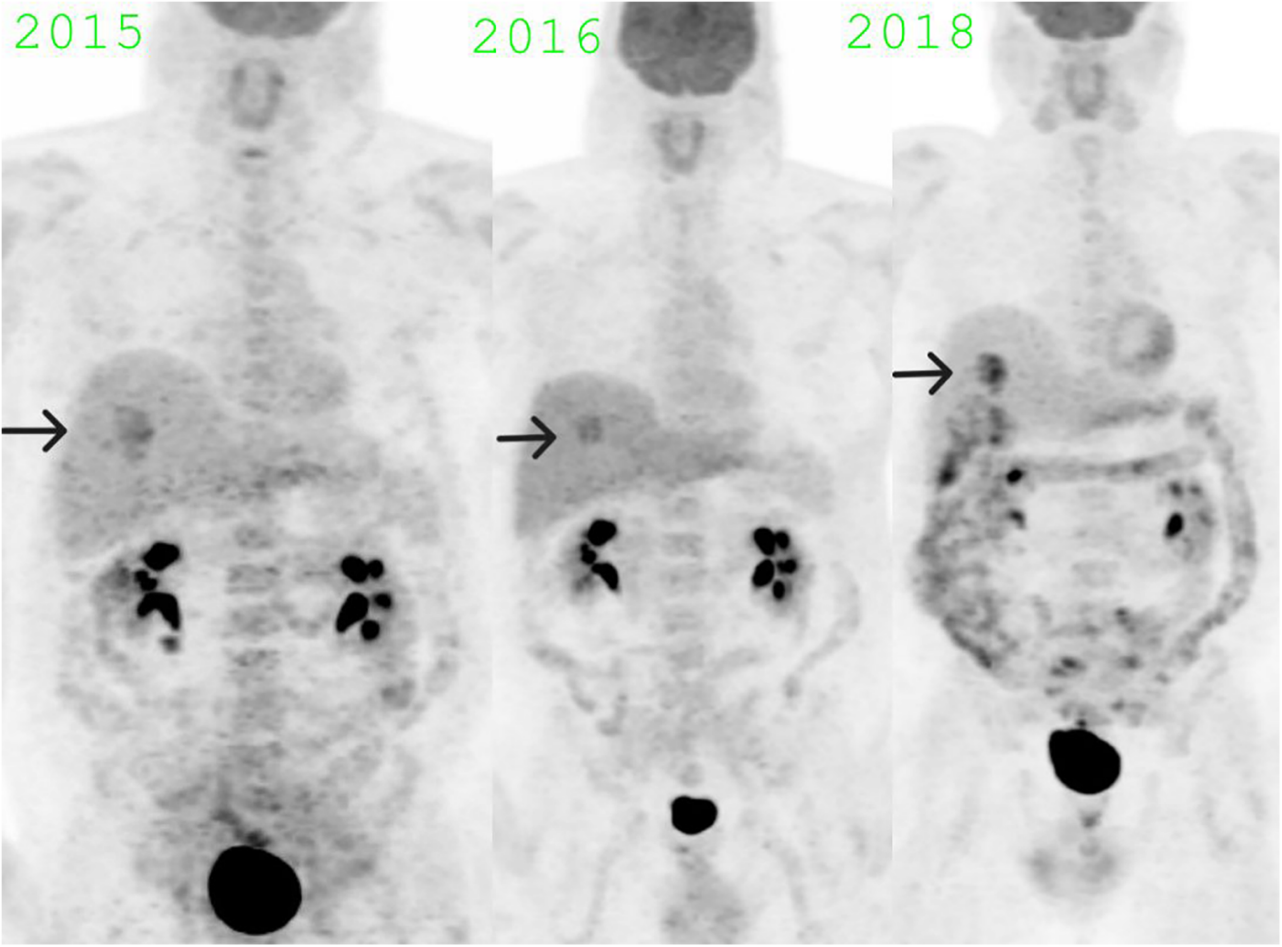
MIP images showing fluctuating SUV max in the hibernoma over the years: SUV max 6.5 in November 2015, SUV max 5.3 in June 2016, SUV max 7.8 in December 2018.

The lack of solid components, stability in the CT appearance over several years, and fluctuating high FDG uptake (Figure 4) made Hibernoma a most likely diagnosis. The presence of vessels within the lesion, imparting heterogeneity on the CT component, further confirmed the diagnosis by differentiating it from a simple brown fat aggregate (5). As of April 2023, the patient is disease-free from melanoma. The physical and scanning parameters for all three PET/CT studies have been tabulated (Table 1).

**Table 1:** Patient FDG PET-CT scan parameters, medication history, and clinical data across multiple scans (2015-2018).

Date/time	Administered patient dose (MBq)	Medications	Weight (kg)	Blood glucose level (Mmol/L)	Fasting since	Background hepatic SUV max	CT parameters (GE medical systems discovery STE)
Nov 2015/15:45	383	Metformin Allopurinol Simvastatin Omeprazole	126	12.6	07:45	3.5	Slice thickness: 3.75 mm KV: 120
June 2016/09:40	394	Metformin Allopurinol Simvastatin Citrazine	125	7.4	Last night	4	Slice thickness: 3.75 mm KV: 120
April 2018/16:40	456	Metformin Allopurinol Simvastatin	124	11.8	10:00	2.8	Slice thickness: 3.75 mm KV: 120

## Discussion

Mammals have two types of adipose tissue: white fat, which stores heat and provides insulation, and brown fat, which is capable of generating heat in response to hypothermia (non-shivering thermogenesis) and food intake (diet thermogenesis) (6). While neonates harbor a significant amount of brown fat, white fat gradually replaces it as they age. Nevertheless, small amounts persist in specific body regions, such as the neck, supraclavicular regions, axillae, retroperitoneum, perinephric area, intercostal spaces, and the thoracolumbar paravertebral regions.

Hibernomas, named for their resemblance to brown fat in hibernating animals (7), are infrequent, benign, slowly growing tumors of brown fat. Initially believed to develop in areas with residual brown fat, subsequent research indicated a higher prevalence in the thighs (30% of cases), with other reported sites being the shoulder, back, and neck (2). In these areas, they may present with swelling or pain or can be asymptomatic with incidental discovery on imaging (8). The unique case presented here shows a hibernoma in the perihepatic area, which has never been reported in the literature before.

Typically appearing in the third to fourth decade of life, these tumors exhibit a varied gender predilection between males and females, as reported in the literature (9). Malignancy is not reported in the literature, and hibernomas are usually asymptomatic, though they may cause neuropathic pain due to nerve compression. In the presented case, the patient had no symptoms specific to the perihepatic lesion.

Macroscopically, hibernomas are partially encapsulated and lobulated, with a cut surface ranging from yellow to tan. Microscopically, they consist of multivacuolated cells with central nuclei and granular eosinophilic cytoplasm, displaying a unique color due to hypervascularity and abundant mitochondria. Asymptomatic cases generally require no treatment. Complete surgical excision is the definitive treatment, as incomplete excision often results in recurrence (10).

On nonfunctional imaging, hibernomas share characteristics with other fat-containing lesions like lipomas and liposarcomas. On ultrasound, they appear as echogenic masses (8). Their attenuation on Computed Tomography (CT) and Magnetic Resonance Imaging (MRI) falls between fat and muscles, imparting heterogeneity to their imaging features. Large feeding vessels may be identified in them (11), appearing as flow voids on MRI. Hibernomas are well-defined, often multiseptated, and exhibit variable post-contrast enhancement. Few other differentials include angiolipomas, hemangiolipomas, and hemangiopeicytomas.

Hibernomas on FDG PET/CT display high metabolic activity due to their rich mitochondrial content. In contrast, lipomas are usually normo-metabolic, and liposarcomas exhibit low FDG uptake (12), although specific SUV max cutoff values are unavailable. Hibernomas can exhibit a wide range of SUVs and have reported values from 1.9 to 20 in the literature (13). Further differentials for hibernomas on PET/CT include other primary soft tissue cancers, metastasis, infections, and pathological lymph nodes. Since hibernomas have a range of benign and malignant lesions in their differential diagnosis, a thorough evaluation is essential. This includes obtaining a detailed history, considering clinical examination findings, comparing with prior imaging, and closely analyzing the lesion's appearance on the CT component to ensure accurate characterization. Due to their potential to yield false-positive results in cancer assessments, understanding hibernomas’ physiology and imaging appearances is crucial for accurately characterizing these fat-rich lesions.

Biopsy has been recommended in some reports in the literature for histological confirmation of hibernomas. Park, Ogura (14), in their report, concluded that Hibernoma cannot be reliably differentiated from liposarcoma based only on PET/CT due to the considerable overlap of SUV in these lesions. It's worth noting that biopsy can lead to bleeding due to the vascularity of hibernomas (15), and FNA may prove insufficient for an accurate diagnosis. Although there is evidence that SUV max of hibernomas fluctuates according to different seasons (14, 16) this would be difficult to implement. We propose a combination of morphological characteristics on cross-sectional imaging and patterns of metabolism on functional imaging to potentially eliminate the need for biopsy and its associated complications.

The metabolic activity of brown adipose tissue is under the control of the sympathetic nervous system, which releases catecholamine norepinephrine that binds to G-protein coupled adrenergic receptors in brown adipose cells. The value of this mechanism in the diagnosis of hibernoma has been highlighted by Ciappuccini, Bardet (3), who demonstrated a dramatic reduction in the FDG uptake of Hibernoma after administering 60 mg of propranolol 1 h before the injection.

Hibernomas have been incidentally identified not only on FDG PET/CT scans but also on imaging studies that utilized various other radiotracers. These include Technetium-99 m tetrofosmin myocardial perfusion studies (17, 18), [^68^Ga]Ga-FAPI PET/CT (19), ^68^Ga-PSMA PET/CT (20) and ^68^Ga-DOTATATE PET/CT (21). These reports illustrate the diverse receptor patterns present on the surface of brown fat cells and the spectrum of intracellular biochemical expressions found within hibernomas. This suggests there is still much to be comprehended about hibernomas beyond what is currently available in the literature.

## Conclusions

Hibernomas are rare brown fat-containing tumors that exhibit high FDG uptake due to brown fat's thermogenic metabolic characteristics. As observed in this study, the perihepatic occurrence of a hibernoma is unprecedented in the existing literature, adding to its uniqueness. Recognizing this entity is of considerable importance to avoid false positive diagnoses of malignancy, which could lead to unnecessary investigations and cause delays in initiating treatment.

## Data Availability

The original contributions presented in the study are included in the article/Supplementary Material, further inquiries can be directed to the corresponding author.
